# Supplementary material for: Preliminary Results of Feasibility and Acceptability of Self-Collection for Cervical Screening in Italian Women
Source: Pathogens. 2023 Sep 17;12(9):1169. doi: 10.3390/pathogens12091169 (PMC10535381; doi:10.3390/pathogens12091169)
Supplement: Supplementary file 1 [file pathogens-12-01169-s001.zip › pathogens-2609090-supplementary.pdf]

Identification Code: ……………………………….

Year of birth: ………………………………………….

Height: …………………
Weight: ………………..

Level of Education

- Elementary school
- Middle school
- High school
- Degree

Civil Status

- Single
- Married
- Divorced
- Widow

Smoking status: Yes/No

Menopause status: Yes/No

Use of contraceptives: Yes/ No

Use of condoms

- Never
- Occasionally
- Only in the past
- Always

Previous history of STIs: Yes/NO

Year of previous Pap-test: ………………………

Accepatability questionnaire:

Q1: Did you find the self-collection instruction clear and understandable? Yes/NO
Q2: Did you find self-collection easy to perform? Yes/NO
Q3: Do you prefer using the vaginal self-collection than the clinician-collection device? Yes/NO

Direzione Igiene e Controllo delle Infezioni Ospedaliere
Via Padre Manzella, 4 - 07100 Sassari
Direzione: 079 228466
Segreterie: 079 228292,079 228291
Fax: 079 228472
Email: igiene@uniss.it
